# A high-resolution probabilistic *in vivo* atlas of human subcortical brain nuclei

**DOI:** 10.1038/sdata.2018.63

**Published:** 2018-04-17

**Authors:** Wolfgang M. Pauli, Amanda N. Nili, J. Michael Tyszka

**Affiliations:** 1 Division of Humanities and Social Sciences, California Institute of Technology, Pasadena, CA 91125, USA; 2 Computation and Neural Systems Program, California Institute of Technology, Pasadena, CA 91125, USA; 3 Northwestern University, Evanston, IL 60208, USA

**Keywords:** Reward, Magnetic resonance imaging, Decision, Brain, Motivation

## Abstract

Recent advances in magnetic resonance imaging methods, including data acquisition, pre-processing and analysis, have benefited research on the contributions of subcortical brain nuclei to human cognition and behavior. At the same time, these developments have led to an increasing need for a high-resolution probabilistic *in vivo* anatomical atlas of subcortical nuclei. In order to address this need, we constructed high spatial resolution, three-dimensional templates, using high-accuracy diffeomorphic registration of *T*_1_- and *T*_2_- weighted structural images from 168 typical adults between 22 and 35 years old. In these templates, many tissue boundaries are clearly visible, which would otherwise be impossible to delineate in data from individual studies. The resulting delineations of subcortical nuclei complement current histology-based atlases. We further created a companion library of software tools for atlas development, to offer an open and evolving resource for the creation of a crowd-sourced *in vivo* probabilistic anatomical atlas of the human brain.

## Background & Summary

High spatial resolution magnetic resonance imaging (MRI) and improved data pre-processing, especially non-linear image alignment techniques^1–4^, have enabled the systematic investigation of contributions of subcortical brain nuclei to human cognition and behavior. This development has led to an increasing need for a high-resolution probabilistic *in vivo* atlas of subcortical nuclei, for at least three reasons. First, several of the relevant subcortical structures are not discernible when averaging structural brain scans across participants in a single study. Second, uncertainty about anatomical labels for subcortical structures hinders comparisons across studies. Third, because of potential spatial inaccuracies in co-registration to standard space, it is critical to maintain probabilistic atlas labels.

To address this need, we improved upon an approach we recently developed to create the California Institute of Technology (CIT168) probabilistic high-resolution *in vivo* atlas of the human amygdala^5^. We significantly expanded this existing atlas by including probabilistic anatomical labels for subcortical nuclei implicated in human executive function^6,7^. In particular, research in rodents and non-human primates has identified a network of subcortical nuclei at the core of reinforcement learning and decision making (for reviews, see refs 8,9). This work suggests that a network of diverse subcortical nuclei may, among other psychological functions, approximate a biological simulacrum of the long short-term memory model^10^, where striatal dopamine release implements a feedback mechanism for mammals to learn based on successes and failures of their interactions with the environment^11,12^. Dysfunctions in these subcortical nuclei have been identified as candidate sources in debilitating clinical conditions that levy high personal and societal costs, including eating-disorders, obsessive-compulsive disorder, and drug-use disorders^13^. However, current scientific understanding of their functions is strongly informed by animal models (for reviews, see refs 14–16). The resulting uncertainty about whether analogous neural mechanisms are conserved in the human brain has undermined progress in identifying specific biological mechanisms and corresponding therapeutic targets^17^.

The existing CIT168 atlas of the human amygdala has already provided a useful research tool for neuroscience studies across domains. Using a symmetric normalization (SyN) diffeomorphic image transform to map preoperative structural scans of epileptic patients onto the CIT168 atlas, it was possible to discover electrophysiological evidence for item-specific working memory activity in the human amygdala^18^. Another study, with the CIT168 atlas as reference, combined electrophysiological, lesion, and functional MRI data, and found evidence that the human amygdala processes both the degree of emotion in facial expressions and the categorical ambiguity of the emotion shown, and that these two aspects of amygdala processing can be most clearly distinguished at the level of single neurons^19^. The present extension of the CIT168 atlas has already been used in a recent functional MRI study, which found evidence for state value prediction errors, in addition to reward prediction errors, in the human substantia nigra (pars compacta), while participants solved a Markovian decision making task^20^.

In summary, we strove to create a probabilistic *in vivo* reference atlas for subcortical nuclei involved in reward learning and decision making, but not included in existing *in vivo* atlases^21–24^. The current delineation provides a finer parcellation of subcortical nuclei, with more accurate external boundary definition than current histology-based atlases. This atlas is especially useful in conjunction with high accuracy registration methods, such as diffeomorphic warping. We intend for these templates and delineations to be an open and evolving resource for future functional and structural neuroscience studies. To this effect, we further created a library of Python tools for the creation of a crowd-sourced *in vivo* probabilistic anatomical atlas of the human brain.

## Methods

We constructed high spatial resolution *in vivo* templates of the human brain by diffeomorphically registering structural magnetic resonance images from 168 typical adults drawn from the Human Connectome Project^25^. Subsequently, we constructed eight validation templates by averaging 84 T1w and T2w warped image pairs selected randomly from the 168 T1w and T2w image pairs in template space^5^. We delineated subcortical nuclei in the left hemisphere of each validation template, averaged labels from all observers and validation templates, then projected the label averages diffeomorphically to the right hemisphere. This resulted in a bilateral probabilistic atlas with minimal left-right observer bias (see subsection "Probabilistic atlas construction" for details).

### Source data

Structural MRI data was drawn from the Human Connectome Project (HCP) S500 subject release^25^. The minimal HCP structural preprocessing pipeline was therefore already applied to all structural imaging data^26^. To maximize SNR in individual data, we included only subjects for which two separate acquisitions for each of the T1w and T2w structural images were available^26^. Application of this inclusion criterion reduced the available sample to 208 subjects. To perform age and sex unbiasing, we balanced the number of male and female subjects at each integer age between 22 and 35 years old, inclusively. This resulted in a final sample size of 168 individuals (84 females and 84 males, mean±sd age in both groups=28.9±3.6 years).

For those researchers interested in the demographics of the original 168 subjects averaged to make the CIT168 templates, the top level of the OSF storage contains a summary table (CIT168_Unrestricted_Demographics.tsv) for this project, listing the HCP identifier, sex and five-year age range for all 168 subjects used to generate the CIT168 templates. Rich demographic and behavioral data for each subject are made available by the HCP and can be tied to the CIT168 cohort through the HCP identifier. Integer ages for individual subjects are considered protected data by the HCP and we cannot release this information here under the Terms of Use agreement. For more detailed, but restricted demographics, the user should apply directly to the HCP.

### Group template construction

We constructed an unbiased group template using diffeomorphic normalization with a joint cost function over T1w and T2w high-resolution 3D images. We used the bivariate symmetric normalization (SyN) algorithm for all registrations performed, relying on the implementation by the Advanced Normalization Toolbox (ANTs v2.1.0) in the antsMultivariateTemplateConstruction.sh script^4^. We constructed initial unbiased seed templates for T1w and T2w volumes by simple averaging across all subjects. This was possible because all volumes were already rigid-body AC-PC aligned (i.e. without linear scaling) by the minimal HCP structural preprocessing pipeline. For each individual brain, we optimized a single diffeomorphic mapping using a joint cross-correlation similarity metric giving equal weights to the T1w and T2w images^27^. As the individual T1w and T2w images were already coregistered during HCP preprocessing^26^, only a single diffeomorphism is required. Other SyN registration parameters used for this bivariate template include: SyN gradient step=0.25, SyN Gaussian regularization *σ*=3.0 voxels^28^, multiscale downsampling factors=(4, 2, 1), affine registration iterations at each downsampling factor=(10000, 10000, 1000), SyN registration iterations at each downsampling factor=(30, 90, 20). The initial unbiased bivariate template was refined iteratively: After each iteration, a new template was created by applying the newly generated affine and diffeomorphic transforms^5^. We terminated this template refinement after four iterations^29–31^. This resulted in an unbiased, AC-PC aligned pair of T1w and T2w group templates, with 700 μm isotropic spatial resolution, subsequently referred to as the CIT168 templates.

### Probabilistic atlas construction

Some of the included subcortical nuclei cannot be readily delineated in individual structural images from the HCP dataset. In contrast, most nuclei become sufficiently well-defined in the group average templates, either directly, or because of well-defined surrounding subcortical structures. This enables manual labeling in group templates generated from approximately 80 or more registered individual structural images. We generated T1w and T2w templates for intra-observer label validation and probabilistic atlas generation by randomly selecting then averaging 84 T1w and T2w image pairs from the original 168 individual image pairs in the group template space. We then created the complementary T1w and T2w validation templates, by averaging over the remaining 84 image pairs. We created eight validation templates in total (four samples of 84 from 168 individuals with complements) because we considered this to strike a reasonable balance between the need for intra-rater validation and total labeling time, which was approximately 4 to 8 h per template per observer.

Region labeling was performed in the left hemisphere only, which is not without precedent in the subcortical atlas literature^32,33^. The probabilistic labels for each subcortical region in the left hemisphere were constructed by simple averaging over all 24 manually labeled volumes since all eight validation templates were constructed in the master CIT168 template space. Left hemisphere probabilistic labels were then mapped diffeomorphically to the right hemisphere using the combination of a reflection about the mid-sagittal plane, followed by an affine and then a diffeomorphic transform ("reflection warp"). This results in an anatomically constrained mapping of all points in the left hemisphere to anatomically equivalent points in the right hemisphere. To generate a bilateral labeling, we then applied this reflection warp to the left-hemisphere probabilistic labels using third-order B-spline interpolation. This approach results in a bilateral probabilistic atlas with minimal left-right observer bias. If there is a consistent error in labeling the left hemisphere by one observer, this bias is also mapped to the right hemisphere label, minimizing any left-right observer bias for that region. The accuracy of reflection warping is addressed in previous work^5^. Critically, this allows for inter-hemispheric asymmetry of subcortical nuclei, in location, shape or volume.

### Region delineation

**Figure 1:**
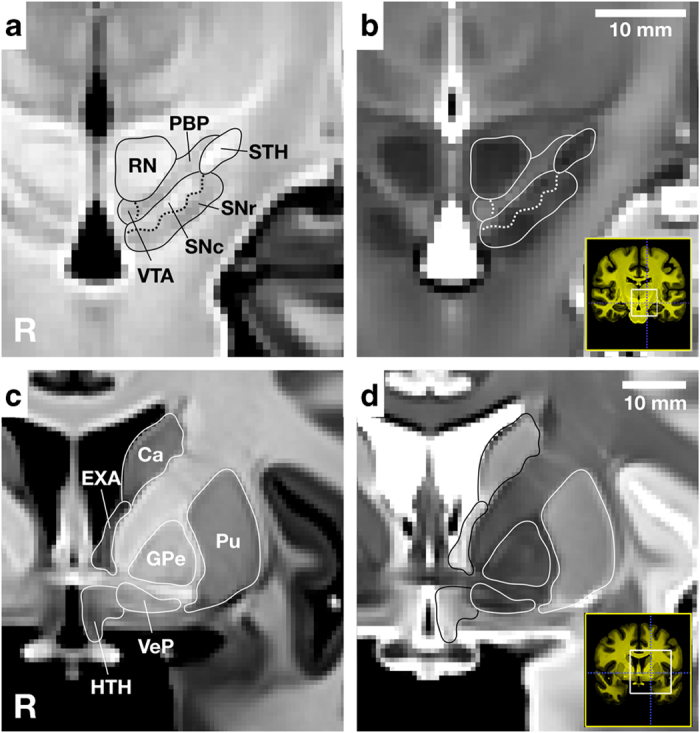
Example tissue boundaries. Example explicit (solid lines) and implicit (dashed lines) boundaries between the red nucleus (RN), parabrachial pigmented nucleus (PBP), substantia nigra (SNc and SNr), and subthalamic nucleus overlayed on the CIT168 T1w and T2w templates. The isotropic voxel size is 700 *μ*m. See Table 1 for label acronyms.

All three observers (AN, JMT and WMP) labeled subcortical nuclei in the left hemisphere in each of the eight validation templates using an agreed upon approach. The primary reference for regional delineation was the Allen Institute Adult 34 year old human atlas (http://atlas.brain-map.org)^34^, with the third edition of the Mai-Paxinos-Voss atlas^35^ as a secondary reference. To allow tissue volumes to be defined by referencing both image contrasts, observers viewed the joint unbiased T1w and T2w templates simultaneously in ITK-SNAP (version 3.6.0)^36^ using a yoked 3D cursor. Tissue boundaries were characterized as either explicit, with clear contrast between neighboring regions, or implicit, where low tissue contrast requires the observer to use surrounding landmarks to estimate the boundary location when labeling (see Figure 1).

The present extension of the CIT168 atlas consists of 16 subcortical gray matter regions labeled by three observers in eight templates. These anatomical labels can conceptually be grouped in the following way. The first group includes subcortical nuclei that are, either directly or indirectly, modulated by dopamine release, and guide behavior^37–39^, including the caudate nucleus (Ca) and putamen (Pu), and downstream areas such as the external and internal segments of the globus pallidus (GPe and GPi, respectively), the subthalamic nucleus (STH), and the substantia nigra pars reticulata (SNr). The second group of nuclei drive activity in the dopaminergic midbrain^6,7,40^ and include the hypothalamus (HTH), habenular nucleus (HN), ventral pallidum (VeP), and the nucleus accumbens (NAC). The third group is comprised of the dopaminergic midbrain regions, including the substantia nigra pars compacta (SNc), parabrachial pigmented nucleus (PBP), and the ventral tegmental area (VTA). The final group consists of landmark structures including the extended amygdala (EXA), red nucleus (RN), and mammillary nucleus (MN).

**Figure 2:**
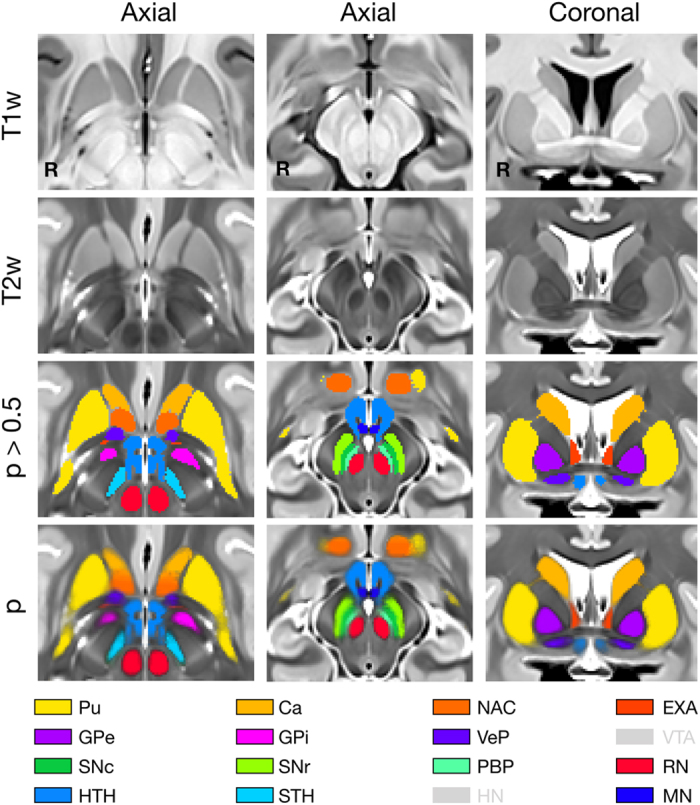
Representative sections from atlas. Axial sections centered on the red nucleus (left column) and substantia nigra (middle column), and a coronal section centered on the ventral pallidum (right column). T1w and T2w sections without overlays show the original tissue contrast seen by observers during delineation. The deterministic (*P*>0.5) and original probabilistic labels are shown in the right two columns. Note the softening of label edges in the probabilistic overlay, indicating inter- and intra-observer variability in boundary location. The color key indicates which labels are visible or out-of-plane (gray).

The regions and the approach to delineation for each are detailed below and the resulting labels illustrated in Figure 2. We invite interested readers to explore the atlas and anatomical labels on the NeuroVault.org website, which supports orthogonal three-plane visualization in the MNI152 space (https://neurovault.org/collections/3145/). The following paragraphs provide a discussion of labeling criteria, as well as their proposed function, with a selective focus on their role within the above computational framework for subcortical contributions to executive functions.

#### Caudate Nucleus (Ca)

The Ca receives strong dopaminergic innervation from the ventral tier of the SNc^41^. It receives synaptic input from prefrontal areas, and also exerts a modulatory influence over prefrontal areas, via the output nuclei (see below) of the basal ganglia^42,43^. The Ca is involved in various goal-directed behavior and cognitive functions^44–46^. The Ca is hypointense in T1w and hyperintense in T2w templates with clear, high contrast boundaries at almost all points. The only exception is at its low contrast ventral boundary with the nucleus accumbens (NAC). The current atlas includes the tail of the caudate as it travels caudally and ventrally around the lateral ventricle.

#### Putamen (Pu)

The Pu receives strong dopaminergic innervation from the ventral tier of the lateral SNc^41^. The Pu is critical for the execution of motor behavior^47,48^. Similar to the Ca, the Pu boundaries are well-defined in both T1w and T2w templates except at the ventral boundary with the NAC. In the current atlas, and in contrast to ref. 35, we include the ventral putamen in the main Pu label, rather than as a separate anatomical label.

#### Globus Pallidus (GP)

The internal and external segments of the globus pallidus are considered to be the output nuclei of the basal ganglia^47,49^. Both the internal (GPi) and external (GPe) segments of the globus pallidus have well-defined margins in both the T1w and T2w templates. In T1w templates, both segments of the GP are hypointense, with clear contrast against the surrounding myelinated white matter, including the internal capsule. The GPe encloses the GPi laterally and rostrally and both lie medial to the Putamen. The anterior commissure (ac) provides a convenient ventral limit for the GPe, separating the latter from the ventral pallidum (VeP).

#### Subthalamic Nucleus (STH)

The tonic activation of neurons of the SNr is at least partially attributable to excitatory input from the STH^47,50^. The STH is a relatively well-defined hyperintense structure in T1w templates and lies caudal and lateral to the hypothalamus. The ventromedial boundary of the STH with SNr and PBP is indistinct in the T2w templates, because all are hypointense. The segmentation of the STH therefore relies on combined evaluation of the T1w and T2w contrast in coronal and sagittal sections.

#### Substantia Nigra, pars reticulata (SNr)

Within the frontal cortical-basal ganglia circuitry^42^, the SNr receives inhibitory projections from the striatum^51^. Neurons in the SNr are tonically active and exert tonic inhibition of the thalamus^51^. Therefore, activation of striatal projection neurons cause a disinhibition of the thalamus, which is thought to produce a gating function^52–54^ for working memory and motor programs in cortical areas^55^. The SNr appears as a hypointense band, ventrolateral to the SNc in coronal T1w sections.

#### Hypothalamus (HTH)

The HTH is involved in a diverse set of functions including hormone release, control of food intake, fear processing, and sexual behavior. Within the scope of reinforcement learning, the lateral HTH is thought to mediate the delivery of rewards^56^, and to cause dopamine release corresponding to a reward prediction error^9^. The hypothalamus is internally heterogeneous in both T1w and T2w templates and is most easily delineated in coronal sections using the anterior commissure, mammillary nucleus, extended amygdala, sublenticular fascicle, and thalamus as landmarks and boundaries. The caudal boundary of the HTH corresponds approximately with the appearance of the RN in coronal sections.

#### Habenular Nuclei (HN)

**Figure 3:**
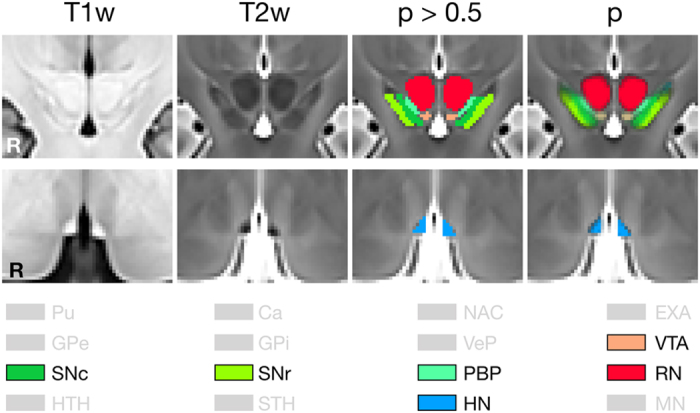
Sections through smaller nuclei. A central coronal section through the VTA, PBP, SNc, and SNr (top row) and axial section through the HN (bottom row). T1w and T2w sections without overlays show the original tissue contrast seen by observers during delineation. The deterministic (*P*>0.5) and original probabilistic labels are shown in the right two columns. The color key indicates which labels are visible or out-of-plane (gray).

The lateral HN has been hypothesized to play a role during aversive learning^57^. The habenular nuclei (or habenula) lie on the medial surface of the dorsal medial nucleus of the thalamus^58^. The HN have been found to exert a modulatory influence over the dopaminergic midbrain^59^. In the context of reward learning, it has been shown to be involved in predicting the exact timing of reward delivery^57,60,61^. The HN have a relatively high myelin content^62^ and are hyperintense in T1w and hypointense in the T2w sections with clear boundaries with neighboring tissue and CSF spaces in all directions (Figure 3).

#### Ventral Pallidum (VeP)

The ventral pallidum is internally heterogenous in both T1w and T2w templates, partially due to its relatively small size, and edge effects with surrounding tissue introduced during midspace template construction (see Methods “Group template construction”). However, it is not entirely indistinct, and can be well localized because of surrounding nuclei. Rostrally, it borders with the nucleus accumbens. Ventrally, it borders with the hypothalamus and substantia innominata. Dorsally, it is separated from the ventral GPe by the anterior commissure (2).

#### Nucleus Accumbens (NAC)

The NAC is bidirectionally interconnected with the VTA and the dorsal tier of the SNc^41^. It thus is both the target of strong dopaminergic innervation, and also modulates dopamine release in dorsal striatal areas^41^. The boundaries between the nucleus accumbens and the caudate and putamen respectively are indistinct, but not entirely invisible and are best delineated using a combination of coronal and axial sections from both T1w and T2w templates. The caudal limit of the NAC coincides with the appearance of the anterior commissure in coronal sections.

#### Substantia Nigra, pars compacta (SNc)

The SNc contains predominantly dopaminergic neurons with projections primarily to the striatum, pallidum, SNr, and STH and plays an important role in a variety of brain functions including motivation, reinforcement learning, and movement control^63^. Prior work has shown an important distinction between a dorsal and a ventral tier within the SNc^41^. However, it is not possible to distinguish these tiers *in vivo* in individual structural images by MRI. The SNc is visible in coronal T1w sections as a semi-continguous, irregularly-shaped, hyperintense band between the PBP and SNr. The rostral limit of the SNc coincides approximately with the caudal limit of the hypothalamus.

#### Parabrachial Pigmented Nucleus (PBP)

Similar to the VTA, the PBP is rich in dopamine neurons^64^, and also projects mainly to the ventral striatum^65^. The PBP is visible as a hypointense band in T2w coronal sections, running ventromedially to dorsolaterally between the red nucleus and the SNc. The PBP has a high contrast, explicit boundary with the hypointense red nucleus dorsomedially and a lower contrast boundary with the SNc ventrolaterally in T2w templates. Rostrocaudally, the PBP extends from the caudal limit of the hypothalamus to the caudal limit of the red nucleus, medial to the subthalamic nucleus.

#### Ventral Tegmental Area (VTA)

The VTA is rich in dopamine neurons, many of these neurons axons terminate in the striatum, so that activation of these neurons results in the release of dopamine in targeted areas^41,65^. The VTA is the target of projections from the shell of the nucleus accumbens^41^, as well as from the central nucleus of the amygdala^66^. In coronal sections, the VTA lies ventral to the RN at the ventromedial limit of the PBP. Rostrocaudally, the VTA extends from the approximate rostrocaudal midpoint of the RN to just beyond the caudal limit of the RN as seen in coronal sections. The boundary with the RN is a well defined and explicit, but the transition from PBP to VTA, and both rostral and caudal limits, are poorly defined and therefore implicit in both T1w and T2w templates (Figure 3).

#### Extended Amygdala (EXA)

The extended amygdala consists of the bed nuclei of the stria terminalis (BNST), the sublenticular extended amygdala (SLEA) and the interstitial nucleus of the posterior limb of the anterior commissure, though this last region cannot be identified in either the T1w or T2w templates and is consequently omitted from the atlas. In coronal sections, the EXA extends ventrally from the ventromedial edge of the caudate nucleus to the anterior commissure, lying between the columns of the forix and the internal capsule (Figure 2). The SLEA extends laterally from the main body of the BNST, immediately caudal to the anterior commissure and dorsolateral to the hypothalamus.

#### Red Nucleus (RN)

The red nucleus is hypointense in the T2w template with clear, high-contrast boundaries with the surrounding midbrain, but has very low contrast in the T1w templates. It is an important landmark for the PBP, SNr, SNc, and VTA labels.

#### Mammillary Nucleus (MN)

The mammillary nucleus is well defined in both T1w and T2w templates with a distinct, more heavily myelinated capsule (comprised mainly of fibers of the mammillary peduncle, fornix and mammillothalamic tract) which appears hyperintense in T1w and hypointense in T2w templates. The MN is readily located at the ventral terminations of the fornix and mammillothalamic tract.

### Volumes of subcortical nuclei

In order to assess the size of the different subcortical nuclei, we estimated the volume of each of the subcortical nuclei by spatial integration of the their voxel-wise probabilities over an entire hemisphere (Table 1). In addition, we calculated a percent laterality index, *L*=(*V*_*i*_-*V*_*r*_)/(*V*_*i*_+*V*_*r*_)×100% (where *V*_*i*_ and *V*_*r*_ are the left and right volume estimates respectively) as a measure of relative inter-hemispheric volume differences for each nucleus.

### Cumulative probability distribution of each atlas label

**Figure 4:**
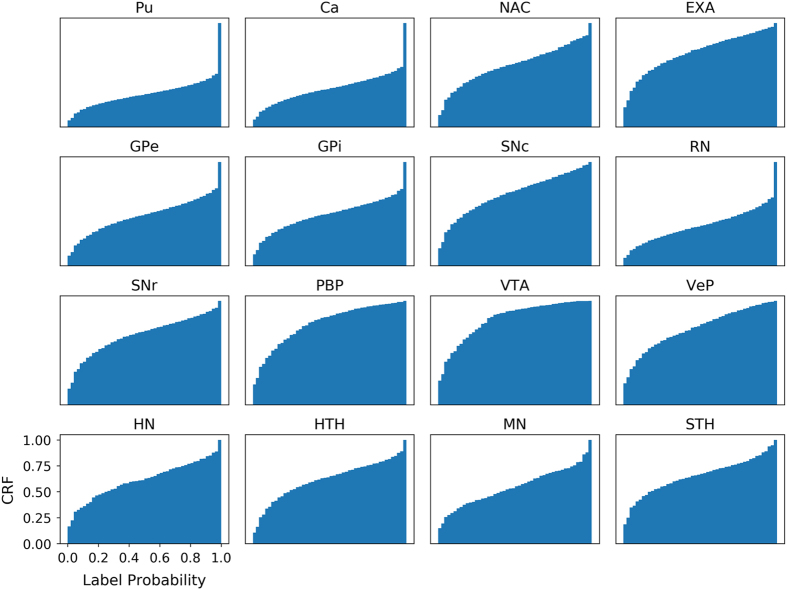
Cumulative relative frequencies of label probabilities. We visualize the uncertainty in defining each subcortical nucleus label by calculating the cumulative relative frequency (CRF) for each probabilistic label over all non-zero voxels. Pu and Ca are examples of labels with a high degree of both inter- and intra-rater similarity, while the more convex cumulative distributions observed for PBP and VTA reflect increased inter-rater variance as the label volume decreases and the tissue boundaries become less reliably defined.

Maintaining probabilistic rather than deterministic atlas labels helps encode observer uncertainty in a natural and well-established way. The cumulative relative frequency distribution of probabilistic atlas labels provides a useful representation of this uncertainty (Figure 4).

### Code availability

Software tools for generating probabilistic atlases from segmented templates and for evaluating inter- and intra-rater reliability are maintained in a publicly available code repository https://github.com/jmtyszka/atlaskit.

## Data Records

The probabilistic atlas, including anatomical images, T1w and T2w templates, as well as segmentations of labels are available at the Open Science Framework (OSF) (Data Citation 1). All imaging data is in compressed Nifti-1 format. We invite contributions by other researchers, in terms of alternative opinions on labeling of included subcortical nuclei, as well as inclusion of additional subcortical nuclei.

## Technical Validation

We assessed intra- and inter-rater labeling reliability between equivalent labels using two distinct similarity measures: 1. the Dice coefficient (also known as the Sørensen-Dice index), and 2. the directed (or forward) Hausdorff distance. Both measures are reported in units to the voxel dimension. We use the standard definition of the Dice coefficient, D, as the ratio of the intersection volume of two labels to the mean volume of the two labels, in the range [0,1]^67^. To calculate the directed Hausdorff distance, H, between two labeled regions, A and B, we first determine for each voxel in A the minimum Euclidean distance to any voxel in B, and then determine the maximum of all these minimum distances. This definition of H is sensitive to the ordering of A and B, and is therefore typically referred to as the directed Hausdorff distance^68^. The distance H is a measure of proximity between two regions which takes account of shape and orientation. The Hausdorff distance finds frequent application in machine vision, in the context of locating a template object within a scene^69^. Full similarity metrics for all observers and templates are provided in the OSF repository (Data Citation 1). Summary statistics for inter- and intra-rater similarity metrics are presented in Tables 2 and 3. Values of H and D were based only on the left hemisphere labels generated directly by each observer in all eight templates.

### Caveats

This atlas relies on averaging *in vivo* data from many different brains to raise the contrast-to-noise ratio between regions sufficiently for delineation. Despite the accuracy of the diffeomorphic registration, some small-scale individual features will be lost during averaging (for example the idiosyncratic SNr/SNc boundary), resulting in boundaries which can no longer be mapped back to individual data exactly. This is an inevitable limitation of group midspace templates and can only be addressed practically by averaging many repeated measurements from a single individual to raise the contrast-to-noise ratio sufficiently for delineations.

#### Similarity metrics

There are limitations to the use of similarity metrics to demonstrate the validity of tissue delineations, since at best, they may only show that all observers are equivalently biased. We report Dice coefficients and Hausdorff distances, because the two metrics provide complementary information about label location, shape, and overlap within and between observers. Dice coefficients are sensitive to the average volume of the two regions being compared: Small errors in overlap begin to dominate, as the average volume decreases. We therefore expect Dice coefficients to underestimate the similarity of small volume labels, in comparison to large volume labels. For example, Dice coefficients for brain masks routinely exceed 0.95 (ref. 70). Hausdorff distances may therefore be the more appropriate similarity metric for small volume labels.

### Alternative approaches to atlas creation

A variety of three-dimensional anatomical atlases of human subcortical nuclei have been constructed using approaches ranging from mapping delineations made in *post mortem* samples, to the use of structural connectivity estimated from diffusion MRI to functional connectivity estimated from resting-state fMRI. A comprehensive list of subcortical atlases in MNI152 space is available at: http://www.lead-dbs.org/.

Regional delineation in histological stained sections has long been considered the gold standard for brain atlas construction in large part due to the cellular resolution of optical microscopy and the versatility of histological staining, including immunohistochemical labeling. S'uch cytoarchitectonic atlases have been mapped to standard *in vivo* MRI templates^71,72^. There are non-trivial hurdles to accurate registration of *post mortem* stained sections with *in vivo* MR images, arising in part from tissue distortion, shrinkage and damage during fixation and sectioning, the collapse of CSF spaces and larger blood vessels *post mortem* and intrinsic differences in tissue contrast between histological sections and *in vivo* MR images.

One of the most widely used subcortical segmentation tools, Freesurfer^73^ has recently added probabilistic labels based on high resolution MRI of *ex vivo* human brain samples of the hippocampal subfields^74^ and amygdala subdivisions^75^. This approach holds tremendous promise for bridging the gap between cytoarchitectonic atlases and *in vivo* labeling, although the number of brains used to generate these delineations is small (10 to 15) and the donor age at death is high (60 to 91 years).

Localization of subcortical nuclei using diffusion MRI has been reported, using both voxel-level diffusion properties and estimated structural connectivity from diffusion tractography. Examples include the nucleus accumbens^76^) and substantia nigra^77^. However diffusion MRI tractography has well-known biases and inaccuracies when estimating structural connectivity from *in vivo* data^78,79^ and will require significant improvements in both data acquisition and analysis to realize its full potential for human brain atlas construction.

Similarly, the use of task-based functional MRI to localize subcortical nuclei has made significant progress in recent years (for a review, see ref. 15). However, in the near term, this approach is limited primarily by the low sensitivity of many fMRI protocols in subcortical areas^80^. This is further complicated by an incomplete understanding of the function of some subcortical nuclei in humans^16^, in part due to the generally strong reliance on forward inference in functional neuroimaging^81^, and the resulting absence of an exhaustive set of localizer tasks.

## Usage Notes

### Suggested usage

The CIT168 atlas is meant as a useful resource for neuroscience studies which would benefit from a high-resolution *in vivo* probabilistic atlas of subcortical nuclei. Its uses include pre-determined ROIs for fMRI studies^20^, measuring individual differences in subcortical volume / morphometry in disease and health^82^, defining seeds for tractography or functional-connectivity^83^, and targets for electrophysiological recordings^18^. The atlas also represents a useful tool for studying brain anatomy, and for clinical applications. Caution should be given when utilizing this atlas for studies on populations of extreme ages, because of the variability of myelination and therefore T2w contrast during early development, as well as age-related increases in selective atrophy^84,85^.

#### Probabilistic and deterministic atlas labels

The probabilistic labels are an estimate of the likelihood that a specific voxel belongs to a given brain region. Strictly, the probabilistic labels are an empirical estimate of the likelihood that an experienced observer will assign a specific voxel to a given brain region. While the atlas labels can be converted to binary masks (by simple thresholding) for subsequent analyses, we recommend using the probabilistic labels as weights for calculations. The main reasons for this recommendation is that several boundaries (e.g. between SNc and SNr) are highly inter-digitated in individual brains, a feature that is entirely eliminated by averaging in the T1w and T2w templates. Consequently, deterministic labels (binary or integer valued) give a false impression of precision of such boundaries when mapped back to individual brains, and should be avoided where possible. Nevertheless, the *atlaskit* GitHub repository contains a script (prob2det.sh) for the creation of binary or deterministic anatomical labels.

#### Standard spaces

T1w and T2w templates and the probabilistic labels are available in standard MNI152 2009c nonlinear asymmetric space in the OSF repository (Data Citation 1) along with the CIT168 to MNI152 affine and SyN transformation data. In addition, the *atlaskit* GitHub repository contains scripts for registering structural images of individual study subjects to the CIT168 atlas.

#### Value of this atlas

The current atlas templates represent the geometry and tissue contrast of the adult human brain *in vivo*. Uncertainty of boundary locations is encoded in the probabilistic labels themselves. Implicit boundaries and structures blurred by averaging will be harder to delineate consistently, both between and within observers, and the probabilistic label value in this region will decrease accordingly. Similarly, high contrast explicit boundaries are more likely to be delineating consistently and the transition zone between tissues will be correspondingly sharp in the probabilistic atlas.

#### Opportunity to contribute

We ask researchers who would like to contribute to this evolving resource to visit the project on the open science framework (OSF) and the associated GitHub collaborative repository (available at: https://github.com/jmtyszka/CIT168-SubCorticalAtlas). Briefly, contributers will be asked (1) to download the validation templates, (2) to fork the above associated GitHub collaborative repository, (3) to label their brain regions of interest in their preferred tool (e.g. ITK-SNAP), to then either (3a) upload the labeling files to the OSF project page, or to (3b) initiate a pull request via the GitHub repository.

## Additional information

**How to cite to this article**: Pauli W. M. *et al.* A high-resolution probabilistic *in vivo* atlas of human subcortical brain nuclei. *Sci. Data* 5:180063 doi: 10.1038/sdata.2018.63 (2018).

**Publisher’s note**: Springer Nature remains neutral with regard to jurisdictional claims in published maps and institutional affiliations.

## Supplementary Material



## Figures and Tables

**Table 1:** Volumes of subcortical nuclei.

Label		Volume (μl)		
	Acronym	Left	Right	Laterality (%)
Putamen	Pu	5224	5196	0.3
Caudate	Ca	4493	4543	−0.6
Nucleus Acumbens	NAC	397	399	−0.3
Extended Amygdala	EXA	134	136	−1.0
Globus Pallidus (External)	GPe	696	678	1.3
Globus Pallidus (Internal)	GPi	383	383	0.0
Ventral Pallidum	VeP	68	74	−4.3
Substantia Nigra (Compacta)	SNc	132	136	−1.5
Substantia Nigra (Reticulata)	SNr	261	269	−1.4
Parabrachial Pigmented Nucleus	PBP	99	98	0.5
Subthalamic Nucleus	STH	135	128	2.9
Ventral Tegmentum	VTA	33	33	0.1
Hypothalamus	HTH	604	617	−1.1
Red Nucleus	RN	301	298	0.5
Mammillary Nucleus	MN	64	62	2.0
Habenular Nuclei	HN	29	27	4.5
We derived the volume of each subcortical nucleus in microliters by spatial integration of label probabilities over each hemisphere independently. Laterality is a measure of left (negative) or right volume bias between the hemispheres (see main text for formal definition).				

**Table 2:** Inter-rater Similarities.

Label Name	Dice Coefficient		Hausdorff Distance (mm)	
	Mean	SD	Mean	SD
Pu	0.94	0.04	2.67	2.12
Ca	0.93	0.05	3.64	3.39
NAC	0.81	0.15	2.11	1.85
EXA	0.75	0.18	2.11	2.07
GPe	0.88	0.09	1.25	1.03
GPi	0.89	0.08	1.11	0.91
VeP	0.69	0.25	1.20	0.97
VTA	0.55	0.35	2.57	2.31
SNc	0.77	0.17	1.61	1.36
SNr	0.79	0.16	1.72	1.40
PBP	0.62	0.28	2.57	2.53
RN	0.92	0.07	0.79	0.61
HTH	0.81	0.14	2.51	2.16
STH	0.84	0.13	1.09	0.95
HN	0.87	0.10	0.69	0.58
MN	0.85	0.12	0.81	0.65
Summary statistics for inter-rater Hausdorff distances and Dice coefficients averaged over all observers and templates. See Table 1 for acronyms.				

**Table 3:** Intra-rater Similarities.

Label Name	Dice Coefficient		Hausdorff Distance (mm)	
	Mean	SD	Mean	SD
Pu	0.95	0.03	2.24	1.15
Ca	0.94	0.03	2.08	1.08
NAC	0.83	0.10	1.80	1.03
EXA	0.78	0.10	1.87	1.28
GPe	0.90	0.06	1.36	0.82
GPi	0.89	0.06	1.21	0.63
VeP	0.70	0.14	1.33	0.76
VTA	0.71	0.13	1.17	0.59
SNc	0.75	0.11	1.71	0.92
SNr	0.80	0.08	1.74	0.97
PBP	0.72	0.13	1.76	0.95
RN	0.93	0.04	0.90	0.43
HTH	0.83	0.07	2.20	1.11
STH	0.82	0.09	1.30	0.74
HN	0.87	0.07	0.81	0.43
MN	0.86	0.08	0.83	0.41
Summary statistics for intra-rater Hausdorff distances and Dice coefficients averaged over all observers and templates. See Table 1 for acronyms.				
